# Role of amino acid metabolism in mitochondrial homeostasis

**DOI:** 10.3389/fcell.2023.1127618

**Published:** 2023-02-27

**Authors:** Qiaochu Li, Thorsten Hoppe

**Affiliations:** ^1^ Institute for Genetics and Cologne Excellence Cluster on Cellular Stress Responses in Aging-Associated Diseases (CECAD), University of Cologne, Cologne, Germany; ^2^ Center for Molecular Medicine Cologne (CMMC), University of Cologne, Cologne, Germany

**Keywords:** amino acid metabolism, mitochondrial homeostasis, TCA cycle, respiratory chain, proteasome, amino acid recycling, lifespan

## Abstract

Mitochondria are central hubs for energy production, metabolism and cellular signal transduction in eukaryotic cells. Maintenance of mitochondrial homeostasis is important for cellular function and survival. In particular, cellular metabolic state is in constant communication with mitochondrial homeostasis. One of the most important metabolic processes that provide energy in the cell is amino acid metabolism. Almost all of the 20 amino acids that serve as the building blocks of proteins are produced or degraded in the mitochondria. The synthesis of the amino acids aspartate and arginine depends on the activity of the respiratory chain, which is essential for cell proliferation. The degradation of branched-chain amino acids mainly occurs in the mitochondrial matrix, contributing to energy metabolism, mitochondrial biogenesis, as well as protein quality control in both mitochondria and cytosol. Dietary supplementation or restriction of amino acids in worms, flies and mice modulates lifespan and health, which has been associated with changes in mitochondrial biogenesis, antioxidant response, as well as the activity of tricarboxylic acid cycle and respiratory chain. Consequently, impaired amino acid metabolism has been associated with both primary mitochondrial diseases and diseases with mitochondrial dysfunction such as cancer. Here, we present recent observations on the crosstalk between amino acid metabolism and mitochondrial homeostasis, summarise the underlying molecular mechanisms to date, and discuss their role in cellular functions and organismal physiology.

## Introduction

Mitochondria are the major sites for energy production. The tricarboxylic acid (TCA) cycle and oxidative phosphorylation (OXPHOS) are coupled to metabolize energy sources for cellular function and survival. The close cooperation of these two processes ensures energy homeostasis under changing environmental and internal conditions. In addition to energy production, mitochondria are also hubs of cellular metabolism. A variety of metabolites from the TCA cycle are essential for cellular signaling and macromolecule synthesis (Martínez-Reyes and Chandel, 2020). There is constant communication between mitochondria and cellular metabolic state. Mitochondria are remodelled in response to metabolic changes (Melser et al., 2015; Ni et al., 2015; Mishra and Chan, 2016; Esteban-Martínez et al., 2017). Mechanisms involved include stress-induced transcriptional responses, adaptation of the ubiquitin-proteasome system, regulation of mitochondrial proteolysis activity, or alteration of key molecules for mitochondrial fusion and fission (Lin and Haynes, 2016; Mishra and Chan, 2016; MacVicar et al., 2019; Yue et al., 2022). Metabolic alterations and mitochondrial dysfunction have been linked to a number of diseases, including cancer and neurodegenerative disorders (Pacheu-Grau et al., 2018; Vasan et al., 2020).

Amino acid metabolism is one of the most important sources for energy production in the cell. The metabolic pathways of amino acids are largely associated with mitochondria (Guda et al., 2007). In the following, we discuss the interactions between amino acid metabolism and mitochondrial homeostasis, focusing on two important metabolic processes in mitochondria, TCA cycle and OXPHOS. We then discuss the effects of amino acid supplementation or restriction on longevity, focussing on the remodeling of mitochondrial homeostasis. We also present recent observations on the potential role of amino acid metabolism in diseases associated with mitochondrial dysfunction.

### Amino acid metabolism is associated with mitochondria

Amino acids are used for protein synthesis or oxidized as energy sources. In addition, amino acids also serve as precursors of many important metabolites that regulate gene expression, post-translational modifications of proteins, cell fate, *etc.* (Martínez-Reyes and Chandel, 2020). Amino acid metabolism relies largely on mitochondrial enzymes. For example, the degradation of BCAAs, i.e., valine, leucine, and isoleucine, occurs predominantly in the mitochondria, with the exception of the first transamination step, which is catalysed in the cytoplasm. Dysfunction of mitochondrial enzymes for amino acid metabolism is causally related to a number of mitochondrial diseases or disorders in humans (Guda et al., 2007). In the following sections, we discuss the functional link between mitochondrial homeostasis and amino acid metabolism and its implications in aging and disease.

### Maintenance of amino acid levels and mitochondrial homeostasis

**FIGURE 1:**
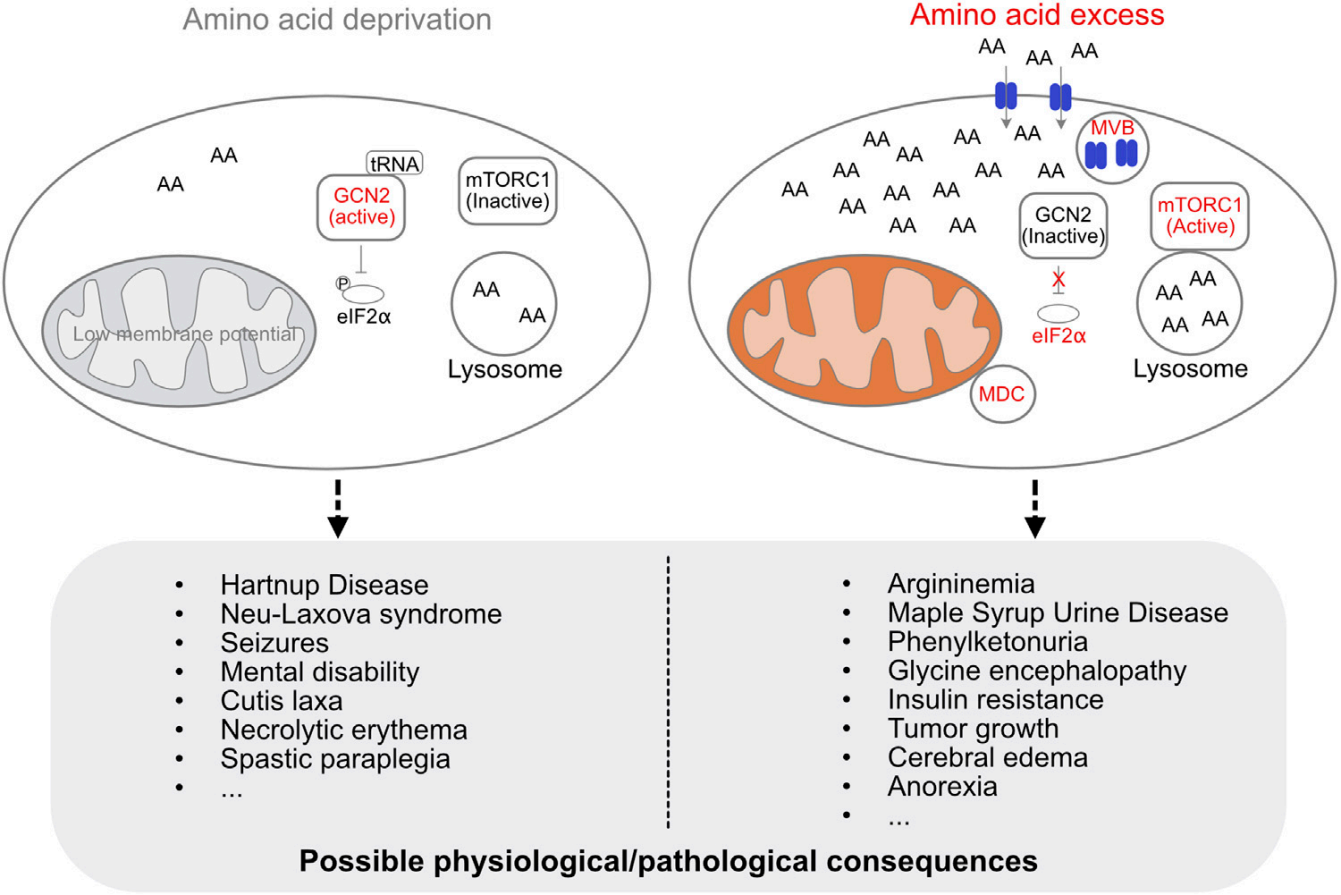
Cellular regulation of amino acid levels and possible physiological/pathological consequences of amino acid deficiency or excess. The lack of amino acids is sensed by GCN2, which is activated upon binding to an uncharged tRNA. Activated GCN2 phosphorylates eIF2α to prevent translation initiation. Amino acid deficiency decreases mitochondrial membrane potential (low membrane potential indicated in grey, otherwise shown in orange). Excess amino acids are imported and sequestered by the lysosome, activate mTORC1, and induce the formation of MDC and MVB. Amino acid deficiency or amino acid overload in the cell can lead to a variety of diseases.

Amino acid levels in the cell are constantly monitored and maintained, and the acquisition, storage, and utilization of amino acids are adjusted according to nutrient status of the cell (Efeyan et al., 2015; Schuler et al., 2021). Amino acid deficiency lowers mitochondrial membrane potential and leads to early onset of mitochondrial respiratory quiescence during oogenesis in flies (Sieber et al., 2016; Yue et al., 2022). The lack of amino acids in the cell is sensed by general control non-derepressible 2 (GCN2), which has a high affinity to uncharged tRNAs. Upon binding to an uncharged tRNA, GCN2 undergoes a conformational change and is activated, resulting in inhibitory phosphorylation of eukaryotic translation initiation factor 2α (eIF2α), preventing translation initiation under amino acid deficiency (Figure 1) (Berlanga et al., 1999; Dong et al., 2000).

Excess amino acids induce the formation of mitochondrial-derived compartments (MDCs) in yeast and mammalian cells (Schuler et al., 2020; 2021). MDCs remove the outer mitochondrial membrane (OMM) protein Tom70 as well as the inner mitochondrial membrane proteins SLC25A carriers, which are known to be major metabolite exchangers *via* the inner mitochondrial membrane (Palmieri and Monné, 2016), for metabolic adaptation during amino acid elevation stress. The yeast vacuole, which corresponds to the mammalian lysosome, serves to import and sequester amino acids to avoid amino acid toxicity (Hughes and Gottschling, 2012; Hughes et al., 2020). At the same time, the endosomal sorting complexes required for transport-dependent multivesicular body (MVB) pathway ensures that nutrient transporters are removed at the plasma membrane along with MDCs and vacuoles to protect cells from amino acid overload (Figure 1) (Schuler et al., 2021). Interesting, leucine appears to be the strongest activator for both MDC formation and mTORC1 activation (Hara et al., 1998; Schuler et al., 2021). In addition, GCN2 knockout mice fed a leucine deficiency diet show more severe effects on new born mouse viability compared with tryptophan or glycine deficiency diets (Zhang et al., 2002). This suggests an important role for leucine in mitochondrial adaptation to nutrient changes. Amino acid deficiency or amino acid overload can lead to a variety of diseases (Koning, 2017; Aliu et al., 2018) (Figure 1).

Sensing of amino acid by mTOR has been shown to contribute to various aspects of aging and disease. For example, mice treated with a special blend of essential amino acids (EAAs) enriched in BCAAs show increased mTOR activity and mitochondrial biogenesis in skeletal muscle and hippocampus, as well as better physical and cognitive performance in aging compared to control-treated animals (Brunetti et al., 2020). Amino acid activation of mTOR has also been linked to the reduction of toxic effects of some chemotherapeutic agents such as doxorubicin by preventing the doxorubicin-dependent mitochondrial damage and oxidative stress (Tedesco et al., 2020). Because activation of mTOR inhibits autophagy, which is essential for the removal of unneeded or damaged proteins and organelles, there is evidence that amino acids may contribute to cognitive decline and synaptic dysfunction at least in part *via* activation of mTOR (Meijer et al., 2015; Wang et al., 2022). In some other cases, e.g., in rats in the maple syrup urine disease model, BCAA administration has been reported to increase autophagy in brain tissue (Fermo et al., 2023). Regulation of mTOR by amino acids also plays an essential role in neuronal development, which was studied recently (Takei and Nawa, 2014).

### TCA cycle and amino acid metabolism

**FIGURE 2:**
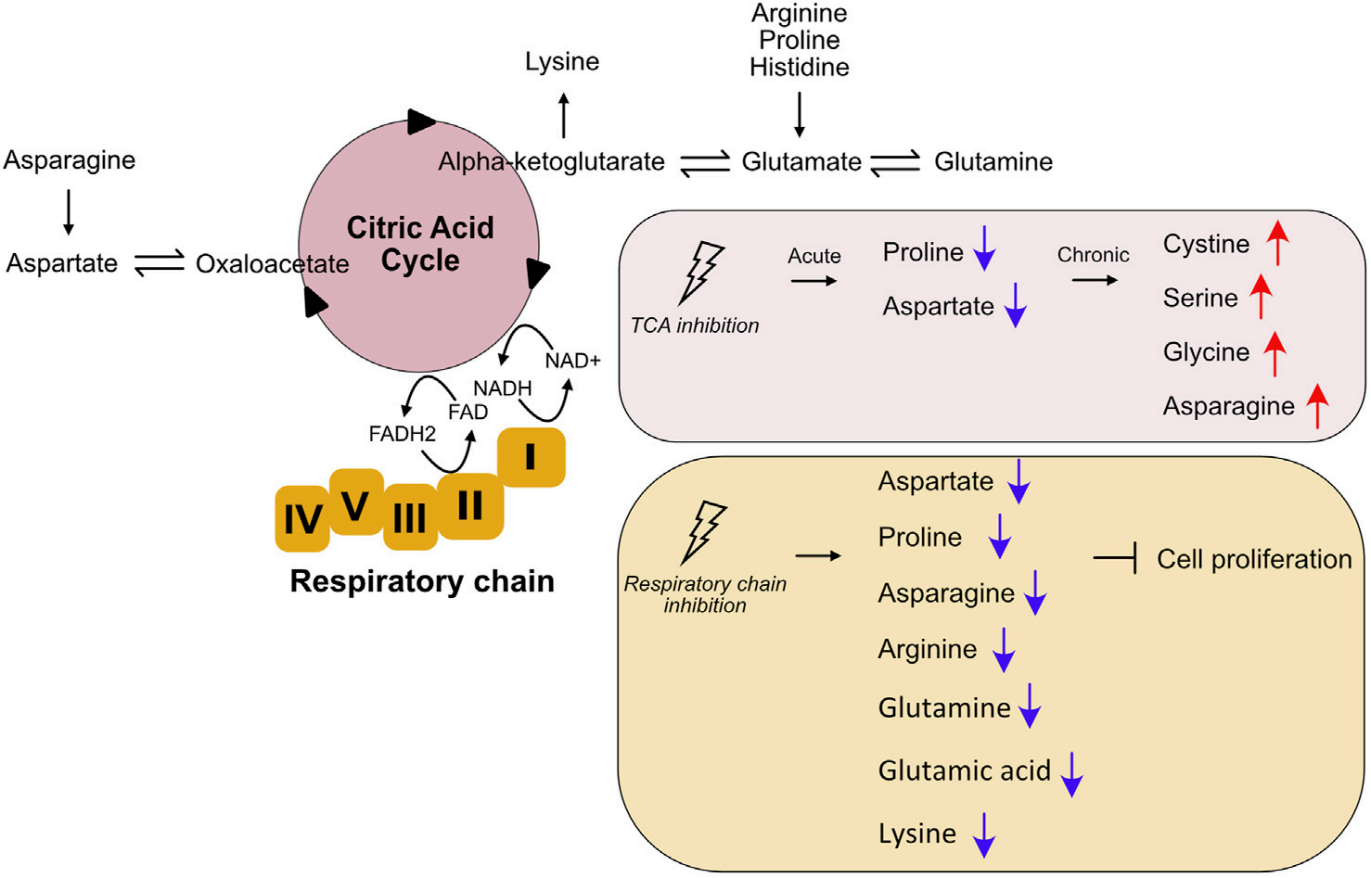
Changes of amino acid metabolism upon TCA cycle and respiratory chain inhibition. The TCA cycle is involved in both catabolism and anabolism of amino acids. The TCA cycle and the respiratory chain are closely linked both physically and functionally. Inhibition of TCA cycle and respiratory chain affect amino acid metabolism.

The TCA cycle is a series of metabolic reactions that occurs in the mitochondrial matrix. The oxidation of fatty acids, glucose, and amino acids produces acetyl-CoA, and the TCA cycle uses acetyl-CoA to produce NADH and FADH2. NADH and FADH2 are then used for ATP production in the respiratory chain. The TCA cycle is not only involved in catabolism, but is also critical for the biosynthesis of a number of molecules including amino acids, lipids, and nucleotide bases (Owen et al., 2002). The components of the TCA cycle serve as precursors for the synthesis of non-essential amino acids, which account for more than 50% of protein carbon (Roberts et al., 1953). For example, the synthesis of glutamic acid and aspartate requires the TCA cycle metabolites alpha-ketoglutarate and oxaloacetate, respectively (Figure 2) (Owen et al., 2002). Detailed regulation and function of TCA cycle has been recently reviewed (Arnold and Finley, 2023).

Although TCA cycle is at the nexus of both catabolic and anabolic metabolism involving different key fuel molecules and metabolites, it was recently reported that alteration of amino acid metabolism is the main consequence of TCA cycle disruption (Ryan et al., 2021). Acute inhibition of the TCA cycle (TCAi) in mouse kidney epithelial cells results in increased glutathione synthesis and impaired proline and aspartate synthesis (Figure 2). Transcriptome analyses of cells with acute TCAi showed robust activation of the response to amino acid deprivation. In addition, there appears to be activation of the integrated stress response in TCAi cells to counteract redox and amino acid stress. Chronic TCAi, achieved by genetic ablation of the two TCA cycle enzymes fumarate hydratase or succinate dehydrogenase, results in increased intracellular cystine, serine, glycine, and asparagine levels (Figure 2). This suggests that chronic disruption of the TCA cycle, as opposed to acute inhibition, may be an adaptive response to metabolic changes. Impaired mitochondrial thiol redox homeostasis causes a similar phenomenon to TCA cycle inhibition, suggesting an important interaction between the TCA cycle, redox biology, and amino acid homeostasis (Ryan et al., 2021). Interestingly, inhibition of different enzymes in the TCA cycle results in different regulation of amino acid metabolism, although common features are evident (Ryan et al., 2021). This suggests that amino acid metabolism is sensitive to specific changes of the TCA cycle and that regulation of amino acid metabolism is adjusted according to this specific change. Since this study is performed in mouse kidney epithelial cells, it would be interesting to see whether these findings apply to different cell types or different tissues, or whether this is conversed across species.

### Respiratory chain activity and amino acid synthesis

Mitochondrial OXPHOS is central to cellular energy production. The OXPHOS system includes five enzyme complexes and two mobile electron carriers (ubiquinone and cytochrome c) that drive the production of ATP (Vercellino and Sazanov, 2022). Similar to the TCA cycle, OXPHOS is not only involved in catabolism for energy production, but also plays an essential role in anabolism, where the synthesis of some amino acids depends on OXPHOS activity as a key to cell proliferation. Inhibition of respiratory chain activity results in auxotrophy for electron acceptors, inhibiting aspartate synthesis and limiting cell proliferation. Addition of pyruvate or alpha-ketobutyrate as exogenous electron acceptors rescues cell proliferation of respiratory-deficient cells. Addition of aspartate restores proliferation in cells lacking an electron acceptor, suggesting that respiratory chain activity is critical for cells to generate aspartate (Birsoy et al., 2015; Cardaci et al., 2015; Sullivan et al., 2015). In addition to aspartate, it has been observed in mouse renal epithelial cells that inhibition of complex III with antimycin A also significantly decreases asparagine and proline content while promoting glutathione synthesis (Ryan et al., 2021).

Mitochondrial respiration in fission yeasts is also required for the provision of amino acids during fermentative propagation (Malecki et al., 2020). Interestingly, supplementation of arginine alone is sufficient for rapid cell growth when the respiratory chain is inhibited, rather than requiring all amino acids. Moreover, not only arginine but also amino acids from the TCA cycle, i.e., glutamine, glutamic acid, and lysine, are required for rapid cell growth when the respiratory chain is blocked (Figure 2) (Malecki et al., 2020). In addition, blocking respiration leads to transient inhibition of TOR, which depends on the AMPK signaling pathway (Malecki et al., 2020). The fact that glutamine, lysine, and glutamic acid are derivatives of alpha-ketoglutarate produced by the TCA cycle suggests inhibition of the TCA cycle when the respiratory chain is inactivated. This is to be expected because the TCA cycle and OXPHOS are both physically and functionally coupled. The utilization of NADH and FADH2 in complexes I and II is required for the TCA cycle to continue to function. There is further evidence of close cooperation between the TCA cycle respiratory chain activity in response to stress. For example, inhibition of respiration leads to a transcriptional switch in the expression of TCA cycle genes in fission yeast to replenish TCA cycle metabolites that support anabolic pathways (Liu and Butow, 1999). Overall, respiratory chain activity plays a key role in supporting amino acid homeostasis and cell proliferation, although the specific mechanisms vary among different organisms studied to date. Coordination between the TCA cycle, OXPHOS, and amino acid metabolism may be an important mechanism for cells to cope with stress conditions.

### Proteasome-mediated recycling of amino acids and mitochondrial homeostasis

Inhibition of the proteasome results in decreased levels of intracellular amino acids and causes mortality. Interestingly, the deleterious effect of proteasome inhibition can be reversed by amino acid supplementation (Suraweera et al., 2012). This finding highlights the important role of amino acid recycling by the 26S proteasome in cellular survival, although it remains unclear how free amino acid content affects cellular physiology. Despite the role of amino acids in protein synthesis, another important aspect is mitochondrial homeostasis due to the close interaction between amino acid metabolism and mitochondria. Amino acid deficiency can directly disrupt the TCA cycle and the respiratory chain, leading to cellular energy failure. In addition, lysosomal recycling of lysine and arginine has been shown to be important for ER quality control (Higuchi-Sanabria et al., 2020). It would be interesting to see whether this is specific to lysosomal function or also applies to proteasome-dependent amino acid recycling. Furthermore, disruption of the mitochondrial respiratory chain or BCAA catabolism impairs proteasomal degradation in the cytoplasm (Segref et al., 2014; Ravanelli et al., 2022). This suggests that mitochondrial activity influences ubiquitin-mediated proteasomal protein turnover, and hence amino acid availability in the cell, which may represent a feedback mechanism for both amino acid metabolism and mitochondrial homeostasis. A recent finding shows that cells lacking ATAD1, an AAA + ATPase that removes substrate proteins directly from the OMM for degradation, are hypersensitive to proteasomal inhibition (Winter et al., 2022). This again points to the importance of the interaction between proteasome function and mitochondrial homeostasis.

Since mitochondria synthesize and degrade proteins independently, the question arises whether the availability of free amino acids affects protein turnover in mitochondria and how it influences the balance between mitochondrial and cytosolic proteins. Imbalance between mitochondrial and cytosolic proteins impairs mitochondrial function (Wang et al., 2021).

### Amino acid metabolism, mitochondrial dynamics and mitochondrial quality control

An interesting aspect yet has been little explored is how mitochondrial dynamics and quality control mechanisms may communicate with amino acid metabolism. Mitochondrial fission and fusion are important mechanisms for metabolic adaptation. OXPHOS activity and oxidative stress are associated with the regulation of mitochondrial fusion *via* modulation of Opa1, an important molecule for inner membrane fusion, while starvation and OXPHOS activity regulate mitochondrial fission *via* modulation of Drp1, a GTPase recruited to OMM to mediate mitochondrial fission (Mishra and Chan, 2016). However, much less is known about how amino acid metabolism may regulate mitochondrial dynamics. Moreover, mitochondrial quality control ensures proper mitochondrial function by removing damaged or unneeded mitochondrial proteins or, at some point, the entire organism (Song et al., 2021). The mitochondrial proteome is drastically remodelled under metabolic stress conditions. A previous study has shown that lipid metabolism regulates the activity of YME1L proteolysis, which remodels the mitochondrial proteome (MacVicar et al., 2019), but whether and how amino acid metabolism is linked to mitochondrial quality control remains to be investigated. Other mechanisms of mitochondrial quality control include mitochondria-associated degradation, mitophagy and mitochondria-derived vesicles (Song et al., 2021). It would be interesting to study how amino acid stress affects the machinery of mitochondrial quality control and its role in adaptation to stress conditions.

### Amino acid metabolism and lifespan regulation

Dietary control or supplementation of amino acids has been shown to affect lifespan in various organisms including worms, flies, and mice. In *Caenorhabditis elegans,* supplementation of individual amino acids, with the exception of phenylalanine and aspartate, extended lifespan (Edwards et al., 2015). Administration of amino acids to complex I or complex II mutant worms revealed that complex I activity is required for complete serine- and histidine-mediated life extension, while complex II activity is required for proline-mediated life extension (Edwards et al., 2015). Supplementation of TCA cycle intermediates also affects lifespan. Treatment with isocitrate, alpha-ketoglutarate, or succinate at optimal concentrations increased lifespan, whereas supplementation of D-malate at a concentration of 1–10 mM decreased lifespan (Edwards et al., 2015). These results suggest that lifespan extension is closely linked to TCA cycle and respiratory chain activity. In *Drosophila*, restriction of the essential amino acids methionine, threonine, histidine, lysine, or BCAAs extends lifespan (Lee et al., 2014; Juricic et al., 2019; Richardson et al., 2021). In rodents, dietary restriction of BCAAs increases mouse health and longevity (Richardson et al., 2021). When dietary restriction mice were fed EAAs but not non-essential amino acids, the effect of dietary restriction-dependent longevity prolongation was reduced (Yoshida et al., 2018). These observations indicate that EAAs have a robust effect on lifespan in flies and mice.

Some studies suggest that high concentrations of BCAAs selectively disrupt mitochondrial pyruvate utilization in a mouse model of ischemic heart injury (Li et al., 2017), while others showed that a BCAA-enriched mixture increased the average lifespan of mice, increased mitochondrial biogenesis and sirtuin one expression in primary cardiac and skeletal myocytes and in cardiac and skeletal muscle of middle-aged mice, and upregulated reactive oxygen species defense system genes (D’Antona et al., 2010). The discrepancy between the effects of BCAA on lifespan needs further investigation. Nonetheless, these data demonstrate an important role of BCAAs in aging through the regulation of mammalian mitochondrial homeostasis. Overall, amino acid metabolism plays an important role in regulating longevity, with mitochondrial homeostasis being at least one of the important mechanisms mediating the effect of amino acids on life expectancy.

### Amino acid metabolism and mitochondrial dysfunction related diseases

Human mitochondrial diseases (MDs) are often associated with metabolic abnormalities. A detailed discussion of MDs has been reviewed recently (Rahman and Rahman, 2018). Here we specifically discuss how impaired amino acid metabolism may be involved in mitochondrial dysfunction and disease. Altered concentration of certain amino acids is observed in a number of mitochondrial diseases and cancer. In addition to alanine, a standard blood biomarker in MDs (Shaham et al., 2010), patients with primary mitochondrial respiratory chain disease also have significantly elevated levels of BCAAs (Clarke et al., 2013). A recent metabolomic analysis of MDs revealed that a multi-biomarker of four metabolites in blood (sorbitol, alanine, myoinositol, cystathionine) is specific for primary MDs (Buzkova et al., 2018). In addition to the observed changes of amino acid concentration, mutations of mitochondrial enzymes for amino acid metabolism lead to severe metabolic diseases (Guda et al., 2007). For example, mutations in the IVD gene encoding the enzyme for leucine degradation cause isovaleric acidemia, in which the intermediate isovaleric acid accumulates and damages the brain and nervous system (Vockley and Ensenauer, 2006). The deleterious effect of accumulated isovaleric acid is closely linked to proteasomal activity (Segref et al., 2014; Ravanelli et al., 2022).

Glutamine metabolism plays an important role in both MDs and cancer due to its versatile role in cell metabolism (Yoo et al., 2020). Cells with an mtDNA mutation have altered levels of glutamine and glutamine metabolites. These cells have a defect in OXPHOS and rely on oxidative glutamine-glutamate-alpha-ketoglutarate metabolism for survival (Figure 2). Alpha-ketoglutarate supplementation improves the survival of mtDNA-mutated cells (Chen et al., 2018). Moreover, glutamate metabolism is altered in a mouse model of mitochondrial myopathy, and alpha-ketoglutarate supplementation rescues amino acid imbalance in muscle. These results suggest that alpha-ketoglutarate supplementation may be a therapeutic strategy for mitochondrial myopathies (Chen et al., 2018). In addition, the important role of glutamine in cell proliferation makes it a target for cancer treatment. Agents targeting glutamine metabolism, such as glutaminase or conversion of glutamate to alpha-ketoglutarate, have been developed (Zhang et al., 2017). Besides glutamine, other amino acids have also been linked to cancer. For example, LAT1 (*SLC7A5*), the major BCAA transporter, is highly expressed in many cancers, including lung cancer, prostate, and breast cancers (Singh and Ecker, 2018; Häfliger and Charles, 2019). The serine transporter ASCT1 (*SLC1A4*) is upregulated in both breast cancer and lung cancer (Pollari et al., 2011; Maddocks et al., 2016). In addition, amino acids are important for fetal development. Decreased placental transfer of essential amino acids is a common feature in human and animal models of intrauterine growth restriction (Ross, et al., 1996; Anderson et al., 1997; Jansson et al., 1998; Paolini et al., 2001). The role of dietary amino acids in reproductive disorders was reviewed recently (Hussain et al., 2020). Alterations in amino acid metabolism have been associated with mitochondrial dysfunction as well as underlying diseases such as cancer and fetal growth restriction syndrome. A better understanding of the communication between amino acid metabolism and mitochondrial homeostasis may be important for new therapies for diseases with mitochondrial dysfunction.

## Conclusions and perspectives

Changes in mitochondrial dynamics and functionality are often accompanied by metabolic adaptions, and cellular metabolic changes in turn lead to mitochondrial remodeling. Metabolism of most amino acids depends on the proper function of mitochondrial enzymes. Metabolic intermediates of amino acids are fed into the TCA cycle, and metabolites from the TCA cycle serve as precursors for amino acid synthesis. Dietary control or supplementation of amino acids has profound effects on the regulation of lifespan in various organisms, including worms, flies, and rodents. These age-related effects of amino acids occur directly or indirectly through the regulation of mitochondrial function. Certain amino acids such as alanine and BCAAs have been shown to be significantly elevated in primary MDs. Rewiring of the metabolic pathways of glutamine and other amino acids is thought to be an important bioenergetic adaptation in OXPHOS-defective cells or cancer cells. Recent studies have highlighted the role of the TCA cycle and respiratory chain activity in coordinating amino acid metabolism and mitochondrial homeostasis. Disruption of the TCA cycle or respiratory chain activity often has a reciprocal effect. Therefore, it is unclear whether many of the observed phenotypes are a direct consequence of inhibition of the TCA cycle or the respiratory chain. This should be considered in future studies. Dietary control or supplementation of amino acids regulates longevity, although the effects on longevity are contradictory in some cases. Dietary supplementation of most amino acids increases lifespan in worms, whereas restriction of essential amino acids extends lifespan in most studies in flies and mice. This may be due to the different preferred pathways of nutrient utilization in different organisms or to the dose and duration of amino acid supplementation. The sensitivity of different tissues to amino acid supplementation may also lead to different effects on lifespan. More detailed molecular mechanisms need to be characterized, particularly mitochondrial restructuring after amino acid treatment. In addition, the short- and long-term effects of amino acid supplementation on mitochondrial physiology, such as mitochondrial morphology and oxygen consumption, will be analysed. Furthermore, mitochondrial mass and metabolic activity vary in different tissues and cell types. Sensitivity to different mitochondrial gene mutations varies in different tissues, sometimes resulting in tissue-specific phenotypes and diseases (Pacheu-Grau et al., 2018). The interaction between amino acid metabolism and mitochondria may also have tissue-specific features that should be further investigated. Finally, amino acid metabolism offers therapeutic potential for MDs, cancer, and other diseases associated with mitochondrial dysfunction.
